# Complete and Resilient Documentation for Operational Medical Environments Leveraging Mobile Hands-free Technology in a Systems Approach: Experimental Study

**DOI:** 10.2196/32301

**Published:** 2021-10-12

**Authors:** MinJae Woo, Prabodh Mishra, Ju Lin, Snigdhaswin Kar, Nicholas Deas, Caleb Linduff, Sufeng Niu, Yuzhe Yang, Jerome McClendon, D Hudson Smith, Stephen L Shelton, Christopher E Gainey, William C Gerard, Melissa C Smith, Sarah F Griffin, Ronald W Gimbel, Kuang-Ching Wang

**Affiliations:** 1 School of Data Science and Analytics Kennesaw State University Kennesaw, GA United States; 2 Department of Electrical and Computing Engineering Clemson University Clemson, SC United States; 3 School of Computing Clemson University Clemson, SC United States; 4 Linkedin Inc Mountain View, CA United States; 5 NetApp Sunnyvale, CA United States; 6 Department of Automotive Engineering Clemson University Clemson, SC United States; 7 Watt Family Innovation Center Clemson University Clemson, SC United States; 8 Department of Emergency Medical Services Prisma Health Richland Hospital Columbia, SC United States; 9 Department of Public Health Sciences Clemson University Clemson, SC United States

**Keywords:** emergency medical services, prehospital documentation, speech recognition software, natural language processing, military medicine, documentation, development, challenge, paramedic, disruption, attention, medical information, audio, speech recognition, qualitative, simulation

## Abstract

**Background:**

Prehospitalization documentation is a challenging task and prone to loss of information, as paramedics operate under disruptive environments requiring their constant attention to the patients.

**Objective:**

The aim of this study is to develop a mobile platform for hands-free prehospitalization documentation to assist first responders in operational medical environments by aggregating all existing solutions for noise resiliency and domain adaptation.

**Methods:**

The platform was built to extract meaningful medical information from the real-time audio streaming at the point of injury and transmit complete documentation to a field hospital prior to patient arrival. To this end, the state-of-the-art automatic speech recognition (ASR) solutions with the following modular improvements were thoroughly explored: noise-resilient ASR, multi-style training, customized lexicon, and speech enhancement. The development of the platform was strictly guided by qualitative research and simulation-based evaluation to address the relevant challenges through progressive improvements at every process step of the end-to-end solution. The primary performance metrics included medical word error rate (WER) in machine-transcribed text output and an F1 score calculated by comparing the autogenerated documentation to manual documentation by physicians.

**Results:**

The total number of 15,139 individual words necessary for completing the documentation were identified from all conversations that occurred during the physician-supervised simulation drills. The baseline model presented a suboptimal performance with a WER of 69.85% and an F1 score of 0.611. The noise-resilient ASR, multi-style training, and customized lexicon improved the overall performance; the finalized platform achieved a medical WER of 33.3% and an F1 score of 0.81 when compared to manual documentation. The speech enhancement degraded performance with medical WER increased from 33.3% to 46.33% and the corresponding F1 score decreased from 0.81 to 0.78. All changes in performance were statistically significant (*P*<.001).

**Conclusions:**

This study presented a fully functional mobile platform for hands-free prehospitalization documentation in operational medical environments and lessons learned from its implementation.

## Introduction

Prehospitalization information provided by the first responders is often essential to subsequent treatment efforts including the accurate assessment of a patient, medical diagnosis, and rationale for treatment decisions in the emergency care settings. A patient record documented in the field promotes a continuum of care, playing a vital clinical role in the subsequent treatment of patients in emergency rooms, trauma centers, or other receiving facilities. Complete and effective documentation of prehospitalization care informs clinicians and staff of presenting vitals and symptoms, the initial assessment of the condition, attempted prehospitalization interventions, and observed response to the interventions [1-3]. Failure to report initial findings and interventions in the field may result in clinical errors such as inadvertent overdose due to duplicate administration of the same medication by paramedic and emergency department physicians [4-6]. However, prehospitalization documentation is a challenging task and prone to loss of information, as paramedics operate under urgent and disruptive environments requiring their constant attention to the patient [6-9].

The US military has demonstrated an ongoing interest in potential technological approaches that enable efficient prehospitalization documentation at the point of injury in advance of a patient’s arrival to a field hospital [10-14]. Adequate prehospitalization documentation plays a critical role in ensuring casualties’ maximal chance of survival in the operational environments [15-19]. In the past, the United States Army Medical Research and Development Command has successfully deployed a PDA-based mobile platform that enabled efficient data entry to the electronic patient record and transmission of patient information through a wireless network [12,13]. A new challenge has arisen from the PDA-based design interrupting the flow of care when entering electronic health record (EHR) data using keyboards or a stylus. The loss of time for direct patient care is often prohibitive in emergency environments as medical personnel have to continuously conduct hands-on interventions for patients to save their life and limb.

Given the necessity of seeking solutions that will not degrade the clinical workflow, technology solutions using automatic speech recognition (ASR) have been explored for hands-free clinical documentation [20-23]. A mobile platform based on ASR technologies has the potential to enable hands-free documentation by extracting medical information from the incoming audio stream without hand-operated input devices. However, the technologies have not yet proven to be reliable in noise-intensive real-world environments in the context of emergency medicine. In military operations, the environment often involves high levels of noise from factors such as blasts, gunshots, and aircraft. It has been well-documented that the performance of contemporary ASR systems is degraded by heavy background noise, leading to more word errors in speech recognition output [24-29]. Moreover, the noise in ASR audio input may result in specific types of word errors in the output text interfering with the documentation when extracting relevant medical information. The existing publicly and commercially available ASR models are optimized for the daily conversation and thus may perform poorly when applied to domain-specific clinical speech [30,31].

ASR consists of multiple components to convert input audio to output text. There are componentwise interventions known to address the listed challenges at a single component level. Recent studies demonstrated acoustic signal processing algorithms that offer improved resilience of ASR to background noise [27,32]. Some studies improved the noise resilience by implementing speech enhancement algorithms for noise filtering in input audio [33,34], while others trained ASR for various noise patterns to improve its robustness against noise [35,36]. There are well-established methods to establish a customized lexicon for a domain of interest so that ASR could better detect domain-specific terms [37-39]. Some research demonstrated solutions to effectively extracting medical information from clinical text containing both semantic and syntactic errors [40,41].

Despite a number of available component-level interventions, it remains unknown how a combination of all these interventions simultaneously affects the overall performance of hands-free prehospitalization documentation in a noise-intensive operational environment. A technology approach encompassing all possible improvements at every process step of the end-to-end solution has the potential to make a substantial contribution to addressing similar challenges in the daily emergency and prehospital clinical practice.

In this paper, we describe the design of our mobile platform for hands-free documentation in the operational medical environment and lessons learned from its use in a simulated environment. The purpose of the study is to perform a systematic evaluation of improvement opportunities for the platform by aggregating and assessing all possible component-level solutions at every process step. The platform was built to extract meaningful medical information from the real-time audio streaming and generate complete documentation before a patient arrives at a simulated field hospital. To this end, the state-of-the-art ASR solutions with relevant component interventions for modular improvement were thoroughly explored. Development of our platform was guided by qualitative research and structured evaluation to identify and address the relevant challenges through modular improvement at every process step of the end-to-end solution. Physician-supervised clinical simulation drills were conducted for the precise assessment of the system performance in the emergency settings.

## Methods

This research was approved by the Institutional Review Boards of Clemson University (Clemson, South Carolina) and Palmetto Health System (Columbia, South Carolina), with secondary review and approval by the US Army Medical Research and Material Command (Ft Detrick, Maryland).

### Qualitative Analysis for Platform Design and Clinical Simulation

Presimulation focus groups and follow-on simulation drill observations were used to assess medical workflow, scope medical information communicated, user requirements during operation, documentation needs, and overall design of platform. Six focus groups were held with 26 individuals across three categories of employment including emergency medical services, transport nurses, and emergency department physicians (Table 1). Focus groups were conducted by trained facilitators using a semistructured interview guide organized to facilitate a workflow discussion of tasks, communication, and documentation strategies as they approach an emergency, the transition to active treatment, and then transition the patient to the next care team. A total of 21 simulation drills were observed over 3 days. Observers monitored their interaction between equipment, verbal communication, and nonverbal communication as they approached the scene, provided active treatment, and transitioned the patient to the next phase of care. Short debriefing interviews were conducted after each drill to gather participant feedback on the process. A postsimulation focus group was also conducted with participants after each day of drills. Data were documented through detailed notes provided on the observation forms and from the two postsimulation drill focus groups (Multimedia Appendix 1).

**Table 1:** Focus group participants.

	Participants, n (%)	Male, n (%)	Female, n (%)	Experience <10 years, n (%)	Experience 10-20 years, n (%)	Experience >20 years, n (%)	Experience unknown, n (%)
Emergency medical services	6 (23)	5(83)	1 (17)	1 (17)	1 (17)	3 (50)	1 (17)
Transport nurses	11 (42)	8 (73)	3 (27)	1 (9)	2 (18)	8 (73)	0 (0)
Physicians	9 (35)	1 (11)	8 (89)	3 (33)	2 (22)	4 (44)	0 (0)
Total	26 (100)	14 (54)	12 (46)	5 (19)	5 (19)	15 (58)	1 (4)

### Hardware Architecture Design

**Figure 1:**
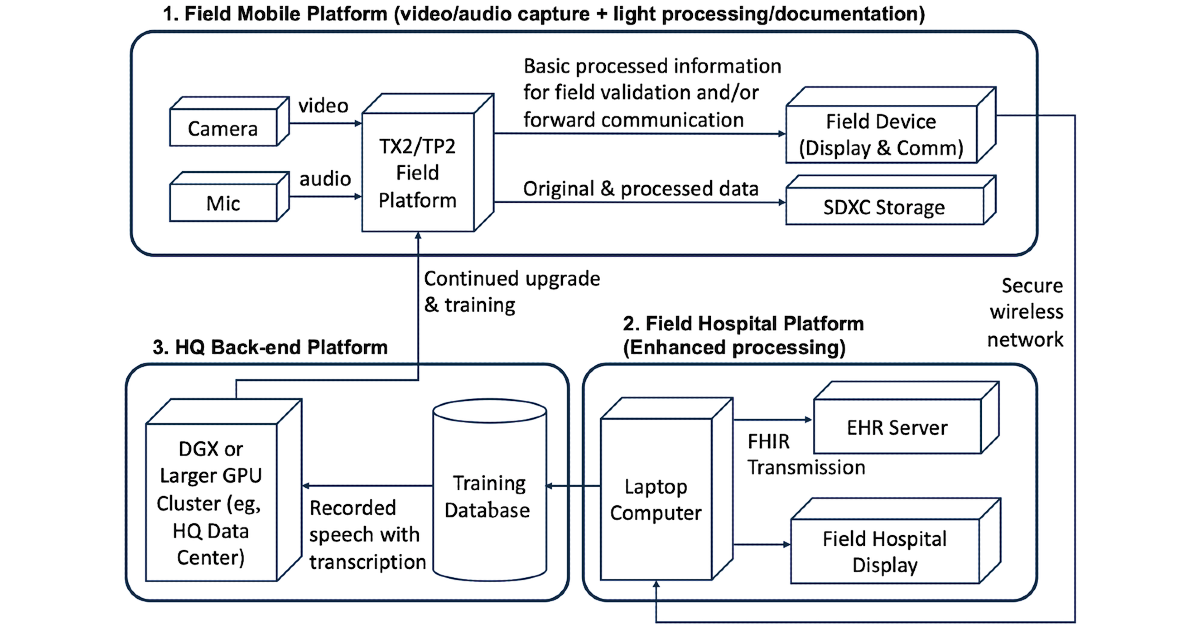
Documentation platform architecture design. EHR: electronic health record; FHIR: Fast Healthcare Interoperability Resources; GPU: graphics processing unit; HQ: headquarters.

**Figure 2:**
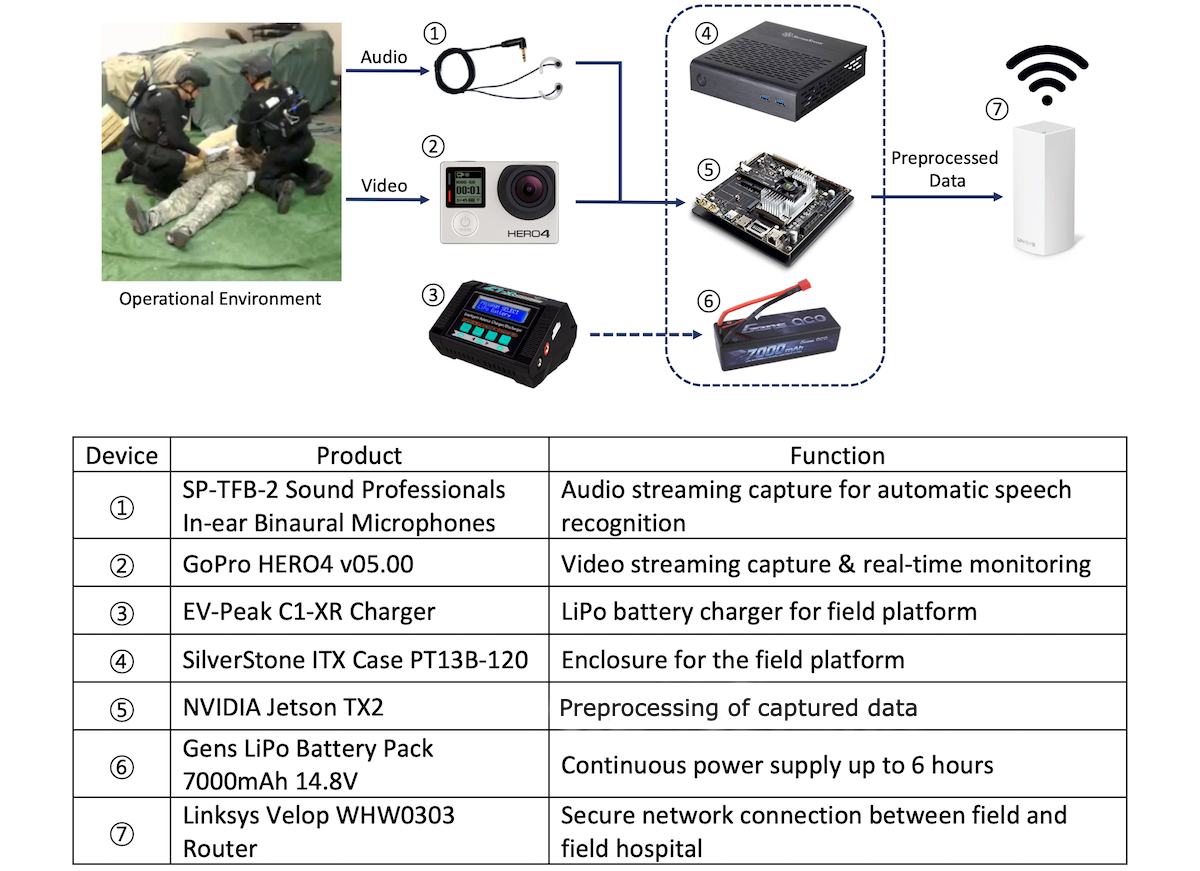
Overview of hardware specifications for field platform.

The overall system architecture design consisted of three major platforms: field mobile platform, field hospital platform, and headquarter back-end platform (Figure 1). The field mobile platform operated on a GoPro camera (video capture), microphone, onboard storage (SDXC memory card), and a mobile form factor graphics processing unit (GPU) system (NVIDIA Jetson TX2; Figure 2). The field mobile platform operated on a 7.4V 7000 mAh LiPo battery, which provided continuous power to the platform for up to 8 hours. The transmission between the field device and the field hospital platform was realized through a closed secure network with multiple Linksys Velop WHW0303 routers under Wi-Fi Protected Access II (WPA2) encryption. The field hospital platform operated on a laptop computer where the received information from the field platform was displayed and converted to Fast Healthcare Interoperability Resources–based data types for improved interoperability with EHR platforms. Dell Poweredge R620 equipped with Cerner Sandbox was deployed as a virtual EHR server throughout the project. The headquarter platform operated on a NVIDIA DGX1 with 8 x NVIDIA Tesla 32GB V100 GPUs and 2 x 20-Core 2.20 GHz Intel Xeon E5-2698v4 central processing units. The ASR training was performed on DGX1 from the headquarter platform, and the output model was downloaded to TX2 in the field platform.

### Software Architecture Design

**Figure 3:**
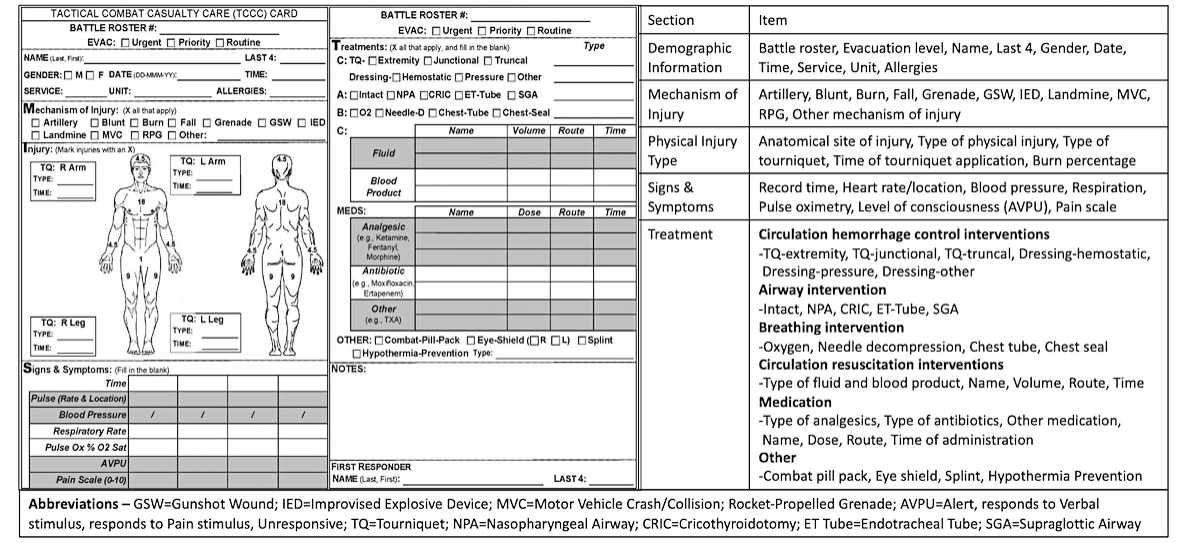
Overview of Tactical Combat Casualty Care card.

**Figure 4:**
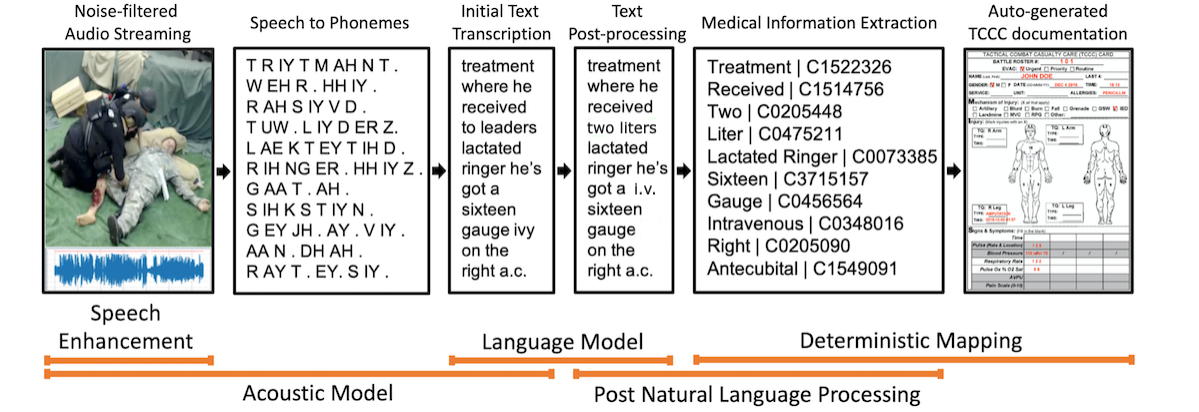
Data processing workflow for hands-free medical documentation. TCCC: Tactical Combat Casualty Care.

The field mobile platform was designed to perform a basic preprocessing of audio captured at the point of injury for the hands-free prehospitalization documentation. The captured audio was converted into a transcript through the ASR module. The Tactical Combat Casualty Care (TCCC) card was selected as the standard format for prehospitalization documentation throughout the study; it has been well-documented that the complete TCCC documentation results in a higher casualty survival rate [11,15]. The ASR output was analyzed to generate bookmarks for the captured video for immediate retrieval of video footages relevant to injuries of interest and to fill out a TCCC card for patients (Figure 3). The captured audio was first passed on to the voice activity detection module, which decides whether the given input is a human voice or not. Next, the audio containing the human voice was processed by a speech enhancement module to emphasize the human voice and minimize background noise (Figure 4). Upon the audio preprocessing, the acoustic model generates the initial transcriptions, which then are corrected and improved through the language model. The language model was designed to infer each word based on its context by using a probability distribution over sequences of words. During the postanalysis, the transcribed text was processed by a post natural language processing module to generate bookmarks for the point of injuries and preliminary documentation of injuries on the TCCC card.

### Modular Improvement for Noise Resilience

The selection of each componentwise intervention was guided by relevant literature and a series of preliminary experiments (Multimedia Appendix 2). A hybrid deep neural network model was used to achieve noise-resilient ASR with its performance comparable to that of the current state of the art. For the implementation of the ASR module, an open-source speech recognition platform, Kaldi, was used for the training of the selected models. A Gaussian mixture model–hidden Markov model was first trained to obtain senones (ie, tied triphone states). Next, the corresponding aligned frames were used for training time delay neural network (TDNN) [42]. The TDNN structure includes an input layer, 11 TDNN layers, and one linear output layer with each TDNN layer set to have 1536 nodes [43]. All weights and biases were discriminatively trained by optimizing the cross-entropy between the target probability and the actual SoftMax output with the backpropagation algorithm [44]. The initial training data consisted of the Switchboard data set (260 hours) and the Common Voice data set (500 hours). Parallel training of the TDNNs using up to 8 NVIDIA Tesla 32GB V100 GPUs was done on the training data with 6 epochs.

**Figure 5:**
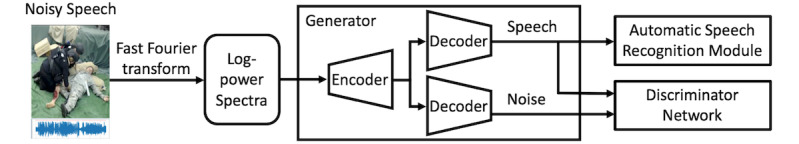
Overview of the generative adversarial network–based speech enhancement architecture.

The speech enhancement module was deployed based on Speech Enhancement Generative Adversarial Network (SEGAN), which enabled the rapid enhancement process without the need for explicit assumptions about the raw data and generalizability to various speakers and noise types [45]. The module was trained using noisy data sets generated by mixing clean data sets with battlefield noise. The original SEGAN has been further improved through log-power spectra-based operation and forked generative adversarial network (ForkGAN) structure to extract both speech and noise information (Figure 5). The ForkGAN architecture operated directly on spectral domain features instead of on raw audio with aims to learn a mapping from the log-power spectra feature input to its feature output, which has demonstrated to outperform other well-known GAN-based speech enhancement techniques [33].

Multi-style training was adopted for additional noise resilience in the operational environment. In specific, ASR was trained with a noisy audio data set containing various types of battlefield noise. A total of 17 battlefield noise files were collected from Signal Processing Information Base [46]. These noises included different types of guns, helicopters, tanks, jets with different speeds, speech babble, and white noise. Additionally, the following other continuous noise types were randomly selected and added to the original training data sets: helicopters, armored vehicles, and tanks. Continuous signal-to-noise ratio values from 0 dB to 20 dB were used to signify different noise power levels. The noisy training data set was created in addition to the original training data sets.

### Modular Improvement for Medical Information Extraction

Our initial investigations showed the original language model was unable to detect medical and military terms used by the medical professionals during the simulation drills. The primary cause of the failure was that these terms were not present in the dictionary that was created from the original ASR training data. To address the issue, a new customized lexicon was trained from medical and military terms used in battlefield-related injuries and medical evacuation. First, the relevant medical and military fields were identified in the TCCC card, the most predominant documentation template of battlefield injuries. Using the Carnegie Mellon University Sphinx Knowledge Base Tool, a dictionary with these domain-specific words and their corresponding phonemes was generated to update the existing language model [47]. The original dictionary and language models were merged with their corresponding new versions, and then the new merged dictionary was compiled to acquire the new lexicon. The Stanford Research Institute Language Modeling toolkit was used to combine the merged language model and dictionary to generate the new grammar model [48]. The new lexicon, new grammar model, and the existing hidden Markov model context-dependency lexicon grammar (HCLG) graph used for the baseline ASR model were combined to construct the updated HCLG graph.

Although all of the aforementioned methods focused on the accurate transcription of conversation between patients and medics, additional processing extracting medical information from the machine-transcribed unstructured text was necessary for completing TCCC documentation. MetaMap is a key tool developed by the National Library of Medicine that has been widely used in biomedical information retrieval and data mining applications to obtain Unified Medical Language System Concept Unique Identifiers (CUIs) with corresponding textual descriptions [49]. The post natural language processing module used MetaMap 2018 for medical information extraction. The following semantic type mappings were configured for the implementation: anatomical abnormality, anatomical structure, antibiotics, body substance, body location, body part, clinical drug, drug delivery device, diagnostic procedure, disease, finding, medical device, quantitative and qualitative concepts, sign, temporal concept, and therapeutic procedure. To prevent excessive false-positive issues [50], a number of sample clinical notes on gunshot, explosion, and head trauma were manually crafted and inputted to MetaMap for identification of the potential CUIs of interest and the corresponding entry location within the TCCC documentation. To clarify, the module was designed for a closed domain application by discarding concepts that are not in the preidentified CUI list. Lastly, the extracted information was automatically entered into the appropriate TCCC sections through a predetermined mapping.

### Clinical Simulation

A total of three clinical simulation drills were conducted in 2017-2019 at Palmetto Health Simulation Center in Columbia, South Carolina. Each physician-supervised drill simulated a typical rescue mission in the medical operational environment. The scope of the simulation spanned from the battlefield to the field hospital, and thus, only the field and field hospital platforms were deployed during the drills. Three common battlefield injury types were used for the clinical scenarios: gunshot wound, amputation due to explosion, head trauma [10,51]. Throughout the drills, all emergency medical care providers taking a role as a medic were wearing the field mobile platform described in Figure 2. The scenarios were loosely scripted by suggesting general descriptions and numbers for vital signs. The medics were allowed to improvise in their verbal reports. The participants acting as patients were also allowed to improvise their responses to medics based on the general description of scenarios.

Each simulation drill started in a room simulating the landscape of field and sky. Various types of battlefield noises were simulated in the room using multichannel high-output speakers. The medics treated patients as they would on a real battlefield during the first encounter. After the initial treatment, the patients were escorted to the flight paramedics waiting at the next meeting point. The patients were then transported to the next room simulating inside of a medical helicopter. Likewise, helicopter noises were simulated in the room using multichannel high-output speakers. After a certain amount of flight time, patients were then transported to an outdoor space where a field hospital had been set up. The patients received the basic examinations at the field hospital, which concluded one simulation drill. The same three clinical scenarios (gunshot wound, amputation, head trauma) were used for each simulation drill in turn. A total of 27 complete patient cases spanning from field to field hospital were simulated and collected, resulting in a total of 5.05 hours of audio recordings. The maximum noise level of 89 decibels was maintained for gunshot and helicopter noise when measured from the patient’s position.

### Overall Performance Evaluation and Statistical Analysis

For qualitative evaluation, thematic analysis using a hybrid inductive and deductive approach was completed in Atlas.Ti 8 (Scientific Software Development GmbH) [52,53]. The analysis process began by reviewing focus group transcripts and observation notes using an open coding format to identify various ways participants described their experiences during different stages within the emergency. This was followed by a round of deductive coding focused on communication strategies and device interaction through the ABCDs of Emergency Care. A final round of axial coding produced four thematic areas. All coding was conducted by one member of the research team.

A standard measure to evaluate ASR performance, word error rate (WER), was used to verify whether the acoustic and language models achieved performance comparable to the current state of the art. However, it was suboptimal to include all conversations captured throughout the drills measure since the main goal of our platform was adequate documentation of injuries rather than transcribing daily conversations. Thus, the primary evaluation measures relied on medical WER and referred to WER for only the sentences from medically oriented speech. For example, sentences from nonmedical conversations between the medics were not considered when evaluating the medical WER. The WER was calculated by comparing machine-transcribed text output and text transcribed by human medical transcriptionists who listened to the audio recording of all simulations. Another primary performance measure was based on the completeness of captured clinical information in the autogenerated TCCC documentation. The captured clinical information was assessed using the F1 score calculated by comparing the autogenerated documentation to the manual documentation by physicians. The cost-effect analysis to identify opportunities for modular improvement was based on how much more clinical information could be captured after each componentwise intervention. McNemar test with Bonferroni correction was used to detect the statistical significance of the improvement effect with respect to medical WER. A total of 4 settings with different combinations of modular improvements were tested using the selected measures. Additionally, one setting based on a commercial ASR solution was assessed using the same performance measures. Dragon Medical Practice Edition 4 (DMPE 4) software (Nuance Communications) is one of the predominant speech recognition solutions that assist clinicians with hands-free voice-dictated documentation in clinics. A setting with its ASR powered by DMPE 4 was compared with the settings with the different modular improvements (Multimedia Appendix 3).

## Results

### Qualitative Study Findings

Four thematic areas include communication methods, communication content, device interaction, and information use (Table 2). Communication methods varied across workflow phases, provider type, and care setting. For example, several focus group participants described frequently using verbal and nonverbal communication strategies with their partner while providing care, and those with military experience discussed this even further. Participants also described situations that they labeled complex communication, whereby they are communicating with and about different patients at one time. This was most frequently discussed as a battlefield experience more so than a transport or field hospital phenomena. Although communication content could vary greatly depending on the workflow phase, the content was remarkably similar within each phase, regardless of the provider type.

Focus group and simulation drill participants’ feedback emphasized the need for device flexibility and for the person wearing it to have control. They also encouraged the design team to make the device strong, durable, and lightweight. Simulation participants recommended that users would have to be trained to use the device and to talk aloud during care so that the device can capture what is being done. Finally, participants shared that short notes and recording that could replace charting would increase user perception of value and thus motivation to use. Physician providers noted that short notes or videos or photos of the injury or emergency site transmitted before patient arrival could be helpful.

**Table 2:** Overview of qualitative study findings.

Theme	Antecedent	Behavior during interaction	Context	Delegation
Communication methods	Use of mnemonics Verbal	More nonverbal To patient To partner	Often chaotic Can dictate if verbal or nonverbal	Must be charted/recorded Very different process at each phase
Communication content	Roles tasks	Only what is necessary If not safe, very little verbal communication Conversely sometimes lots of content at same time—chaotic	Dictates depth/detail Sound an issue for some settings	Preference for who provides hands-off by provider type Content is same at each phase of delegation
Device interaction	Ability to turn on and off prior to hot zone	Cannot get in the way	Flexible locations for different types of providers—helmets, chest, shoulder, etc	When/how to turn off device
Information use	Planning and preparation	N/A^a^	N/A	Help next team

^a^N/A: not applicable.

### Modular Improvement With Componentwise Interventions

The total number of 15,139 words necessary for completing TCCC documentation were identified through transcription from audio recordings collected from all simulation drills. The field mobile platform equipped with baseline ASR achieved a medical WER of 69.9% with 10,582 word errors of 15,139 words (Table 3). Multi-style training incorporating both clean and noise-injected training data sets improved medical WER by a 26.9% decrease in the error rate from 69.9% to 43.0%. The updated language models further reduced medical WER to 33.3%. Although the multi-style training and updated language model decreased the medical WER, deployment of the speech enhancement module increased the error rate to 46.3%. All increases and decreases in the medical WER with the componentwise intervention were statistically significant. The participating physicians identified a total of 768 unique CUIs relevant to the TCCC documentation of gunshot wounds, amputations, and head trauma on the battlefield. The field mobile platform equipped with baseline ASR achieved an F1 score of 0.61. Upon the deployment of the multi-style training, the F1 score increased by 0.11 to 0.72. The updated language models further improved the score to 0.81. However, the score decreased to 0.78 with the deployment of the speech enhancement module.

Among all the componentwise interventions, the combination of multi-style training and an updated language model resulted in the most improvement in medical WER; the error rate was reduced by 36.6% when compared to the baseline model. For specific examples of improvement made by the updated language model, see Table 4. The autogenerated TCCC documentation from our best model (baseline + multi-style training + new language model) achieved an F1 score of 0.81 with 559 true positives, 119 false positives, and 137 false negatives.

**Table 3:** Automated transcription and documentation performance by different settings.

Setting	ASR^a^ transcription output			Automated TCCC^b^ documentation		
	Medical word error rate (%)	*P* value^c^	Precision		Recall	*F*_1_ score
Setting 1: baseline	69.9	N/A^d^	0.484		0.828	0.611
Setting 2: baseline + multi-style training	43.0	Setting 1 vs 2: <.001	0.634		0.824	0.717
Setting 3: baseline + multi-style training + updated language model	33.3	Setting 2 vs 3: <.001	0.803		0.824	0.813
Setting 4: baseline + multi-style training + updated language model + speech enhancement	46.3	Setting 3 vs 4: <.001	0.747		0.819	0.781

^a^ASR: automatic speech recognition.

^b^TCCC: Tactical Combat Casualty Care.

^c^McNemar test with Bonferroni correction was used to calculate the statistical significance.

^d^N/A: not applicable.

**Table 4:** Example of domain-specific word correction with updated language model.

Original language model	Updated language model
air movement *by literally*^a^	air movement *bilaterally*
exit as a *poster here*	exit as a *posterior*
patient is *take kid nick*	patient is *tachypneic*
take *nor vast* for hypertension	take *norvasc* for hypertension
with *pebble radio balls*	with *palpable radial pulses*
active *orchard real* bleeding	active *arterial* bleeding
*ten planting numbering preparation*	*tympanic membrane perforation*
*michael grams offend a nil*	*micrograms of fentanyl*
*full toxins* ninety eight percent	*pulse oximetry* ninety eight percent
soldier triggered *naive do*	soldier triggered *an I.E.D.*

^a^Italics indicate the change between models.

## Discussion

### Performance and Lessons Learned

The previous studies on the extraction of medical information from the human-written clinical text have reported F1 scores ranging from 0.757 to 0.872, depending on a target entity to be recognized [53,54]. Our platform achieved the comparable F1 score of 0.81, despite the multiple challenges posed by errors that are attributed to the machine transcription under noise-intensive operational environments. Our experience deploying the mobile platform has given us four lessons that may be useful in the development of other similar platforms for speech to patient record applications.

#### Lesson 1: Closed Domain Strategy

The observation made by the focus group identified considerable similarity between all patient transportation processes regardless of injury types. For example, all medical personnel described a similar set of information that is expected to share as they transition the patient from one setting to the next. The identified similarity between the processes enabled the labor-intensive closed domain solutions for the post natural language processing without concern for resource constraints (eg, physician time). In our experience, both language model and medical information extraction could be further improved through rule-based or manual tasks such as observation-driven lexicon updates and preidentification of relevant CUIs for reducing false positives. The qualitative study to identify the similarity may provide the basis for cost-effect analysis to examine the feasibility of similar closed domain strategies.

#### Lesson 2: User Training

The importance of user training was pointed out during the focus group study. Accordingly, users were trained to turn on and off the system whenever appropriate, which could prevent the potential false positives incurred by nonclinic conversation. Next, the users were also trained to repeat the information whenever possible. It was observed that, if the same information is repeatedly spoken by a user, the system has a higher chance for complete documentation by properly capturing the information at least once, resulting in the improved high F1 score despite a relatively high WER. We have learned that the proper user training may result in performance improvement as significant as state-of-the-art componentwise interventions.

#### Lesson 3: Impact of Speech Recognition

The performance of the ASR module had a direct impact on the quality of the autogenerated documentation in our speech-to-patient-record application. It was observed that improvement in medical WER after each componentwise intervention is likely to improve autogenerated documentation quality evaluated by the F1 score. As expected, more medical word errors in ASR-transcribed text interfered with the post–natural language processing to extract medical information for documentation. A preliminary observation on the autogenerated documentation revealed that missing words in ASR output and incorrect negation due to word errors were the major causes of false negatives and positives, respectively.

#### Lesson 4: Context of Componentwise Intervention

To some extent, our mobile platform resembles a personal artificial intelligence assistant platform on the commercial market, as it listens to its user and executes desired actions (ie, documentation). Although our platform could deploy the same types of componentwise interventions known to be effective for the commercial platforms, not all interventions were effective in our application. In the context of everyday life, the personal assistance platform can benefit from speech enhancement that emphasizes the speech of the primary speaker (eg, owner of device) while suppressing the speech of secondary speakers. However, in the context of medicine, the same speech enhancement module may cause a higher medical WER by filtering out the patient’s response to doctors or speech from other care providers attempting to deliver information to the primary speaker. Our experience of the performance degradation reveals a necessity for more context-sensitive training for speech enhancement modules to enhance speech from both primary and secondary speakers in the emergency care settings.

Our field mobile platform used only verbal communications for the documentation. As documented in qualitative study findings, information extraction from nonverbal communication along with the verbal communication is essential to reducing the loss of information. Future research may incorporate the existing computer vision solutions to examine if additional information can be extracted from nonverbal communication for more resilient documentation. In response to lesson 2, future studies are warranted to perform a hypothesis-driven study to assess the effect of user training on the resilience of documentation. Lastly, our platform was designed for the closed domain application exclusively for the three most common injury types on the battlefield. Although our study demonstrated that the closed domain strategy can be developed to significantly improve speech recognition performance for the target medical conditions, future speech-to-text and medical information extraction modules may explore to expand the platform design for more variety of medical conditions.

To the best of our knowledge, this was the first attempt to create a fully functional platform for hands-free prehospitalization documentation in operational medical environments. Our application contributes to the body of existing knowledge for the development and assessment of platforms to enable hands-free clinical documentation in real-world noisy environments. The development of our platform was strictly guided by domain experts and a series of structured evaluations to examine modular improvement at every process step of the end-to-end solution. The lessons learned suggest potential refinements in the future endeavors to develop other similar platforms for speech-to-patient-record application.

## Appendix

Multimedia Appendix 1 Complete and Resilient Documentation simulation drill observation form.

Multimedia Appendix 2 Preliminary experiments using a standardized testing data set to guide the selection of componentwise interventions for modular improvement.

Multimedia Appendix 3 Comparison of performance between the presented platform and commercial clinical voice recognition software.

## Abbreviations

ASR: automatic speech recognition
CUI: Concept Unique Identifier
DMPE 4: Dragon Medical Practice Edition 4
EHR: electronic health record
ForkGAN: forked generative adversarial network
GPU: graphics processing unit
HCLG: hidden Markov model context-dependency lexicon grammar
SEGAN: Speech Enhancement Generative Adversarial Network
TCCC: Tactical Combat Casualty Care
TDNN: training time delay neural network
WER: word error rate
WPA2: Wi-Fi Protected Access II
