# Supplementary figures and images for: Complete and Resilient Documentation for Operational Medical Environments Leveraging Mobile Hands-free Technology in a Systems Approach: Experimental Study
Source: JMIR Mhealth Uhealth. 2021 Oct 12;9(10):e32301. doi: 10.2196/32301 (PMC8548972; doi:10.2196/32301)

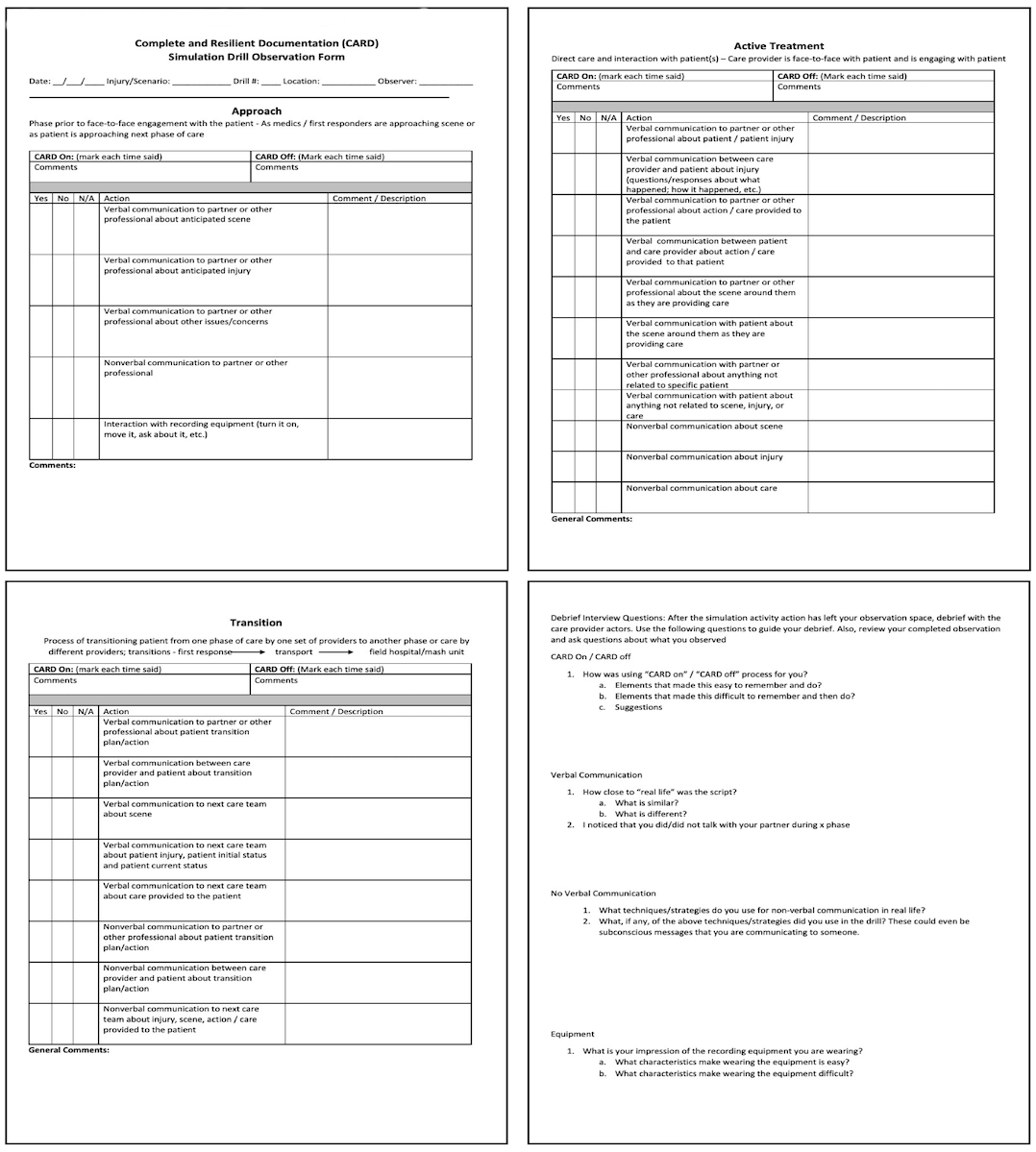

Supplement: Multimedia Appendix 1 [file mhealth_v9i10e32301_app1.png]

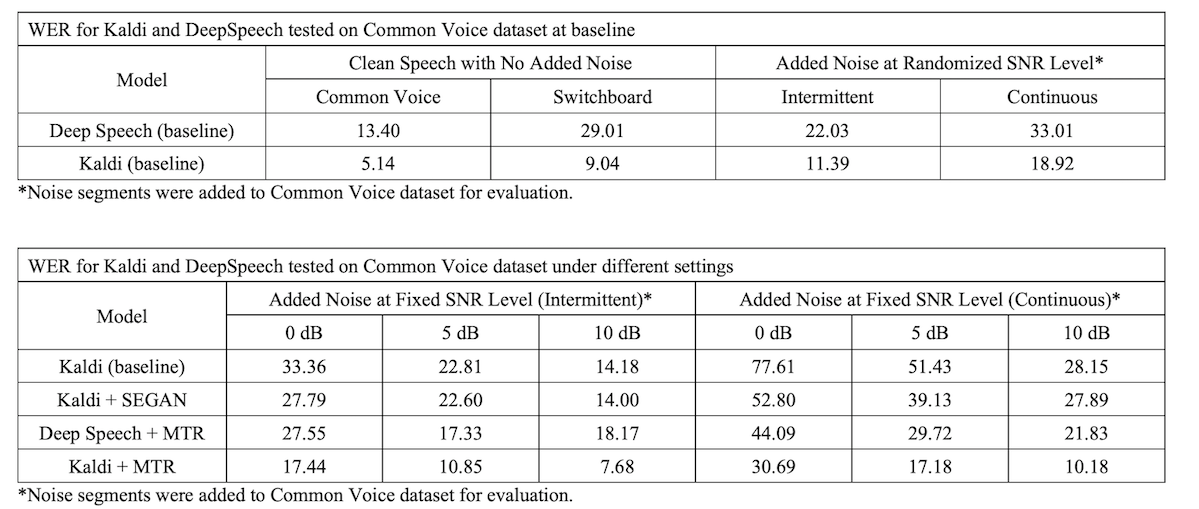

Supplement: Multimedia Appendix 2 [file mhealth_v9i10e32301_app2.png]

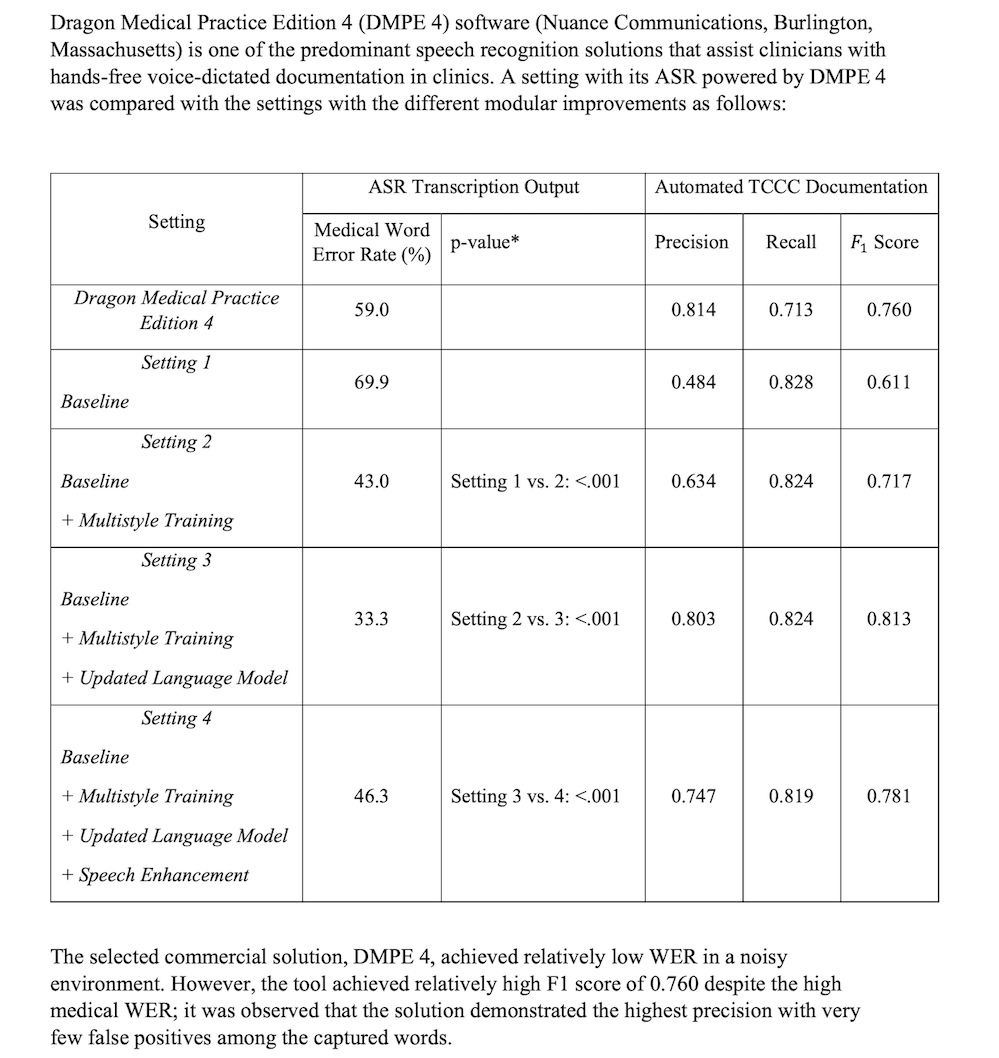

Supplement: Multimedia Appendix 3 [file mhealth_v9i10e32301_app3.png]
